# Complete genome sequence of a HPV31 isolate from laryngeal squamous cell carcinoma and biological consequences for p97 promoter activity

**DOI:** 10.1371/journal.pone.0252524

**Published:** 2021-08-25

**Authors:** Yuri Munsamy, Riaz Y. Seedat, Tumelo R. Sekee, Phillip A. Bester, Felicity J. Burt

**Affiliations:** 1 Division of Virology, Faculty of Health Sciences, University of the Free State, Bloemfontein, South Africa; 2 Department of Otorhinolaryngology, Faculty of Health Sciences, University of the Free State, Bloemfontein, South Africa; 3 Department of Otorhinolaryngology, Universitas Academic Hospital, Bloemfontein, South Africa; 4 Division of Virology, National Health Laboratory Service, Universitas, Bloemfontein, South Africa; Istituto Nazionale Tumori IRCCS Fondazione Pascale, ITALY

## Abstract

Human papillomavirus type 31, although detected less frequently than HPV types 16 and 18, is associated with head and neck squamous cell carcinomas. Previous studies suggest that polymorphisms in the long control region (LCR) may alter the oncogenic potential of the virus. This study reports the first complete genome of a South African HPV31 isolate from a laryngeal squamous cell carcinoma. Sequence variations relative to the HPV31 prototype sequence were identified. The pBlue-Topo® vector, a reporter gene system was used to investigate the possible influence of these variations on the LCR promoter activity *in vitro*. Using mutagenesis to create two different fragments, β-galactosidase assays were used to monitor the effect of nucleotide variations on the p97 promoter. Increased β-galactosidase expression was observed in mutants when compared to the South African HPV31 LCR isolate. Enhanced transcriptional activity was observed with the mutant that possessed a single nucleotide change within the YY1 transcription factor binding site. In conclusion, sequence variation within the LCR of HPV31 isolates may have a functional effect on viral p97 promoter activity.

## Introduction

Human papillomaviruses (HPVs) include over 200 different types and infection by one of the 13 high-risk types increases the risk of developing cervical, anogenital, or head and neck carcinomas (HNSCCs) [1, 2]. These small double-stranded circular DNA viruses of approximately 8 000 base pairs (bp) contain between eight to nine open reading frames (ORFs) [3]. The E6 and E7 genes encode the HPV oncoproteins that promote cell cycle progression and viral DNA replication [3]. The long control region (LCR) contains the viral promoter, p97 and transcriptional elements that regulate expression of the viral oncogenes, as well as transcription factor binding sites and the viral origin of replication [3]. HPV types are distinguished by a genetic difference of around 10% in the L1 gene sequence of the genome [2].

Whilst HPV16 and HPV18 account for the majority of HPV-associated HNSCCs, the contribution of other oncogenic types (31/33/45/52/58) to causing disease should not be ignored [4]. Although the virus is genetically relatively stable, there is differentiation into genomic variants. At the variant lineage and sublineage level, there are nucleotide differences of 1–10% and 0.5–1.0%, respectively [5, 6]. HPV16 is the closest relative of HPV31, however there are differences in carcinogenicity between these two types. HPV16 is identified more frequently in invasive cervical carcinoma as compared to normal cytology than HPV31, with a relative ratio of 3.07 for HPV16 as compared to 0.49 for HPV31 [4].

HPV31 is divided into three major variant lineages and seven sublineages, including sublineages A1, A2, B1, B2, C1, C2, and C3 [8]. Across these HPV31 sublineages, carcinogenicity and viral persistence differs, ranging from HPV16-like to less aggressive behaviour [7]. Nucleotide divergence influences the transcriptional activity of HPV31 LCR variants, which may result in varied oncogenic potential through altered expression levels of the E6/E7 oncogenes. Recently, functional analysis of HPV31 LCR variants showed that polymorphisms associated with lineage C had the greatest ability to alter the expression levels in a reporter gene system, showing a quadrupled increase of expression levels when compared to the prototype [8, 9].

Currently no complete genomes of HPV31 isolates from HNSCCs are available on GenBank. To address our research interests, the complete genome of an HPV31 isolate from a laryngeal squamous cell carcinoma was determined by NGS and the isolate characterised (Genbank Accession nr MW814876). A reporter gene system was used to determine if mutations identified in the noncoding LCR of an HPV31 isolate, had an influence on biological activity. These mutations were sequentially introduced into a reporter vector expressing β-galactosidase activity in a mammalian cell culture system to determine differences in transcriptional activity.

## Materials and methods

### Sample preparation and next generation sequencing

A laryngeal biopsy collected from a patient in the South African province of the Free State that was histologically confirmed as a squamous cell carcinoma with a positive p16 stain was submitted for testing for HPV. HPV was detected using the multiplex PGMY primers targeting the L1 gene [10, 11]. The isolate was designated VBD13/14 and genotyped as HPV31 using bi-directional DNA Sanger sequencing and alignment with GenBank sequence data.

To obtain the complete viral genome, overlapping fragments were generated using primer pair HPV_31F1; HPV_31R1 (5’-GTCCCAAATGGTACAATGGG-3’; 5’-TTCACCAACCGTGCCTGATC-3’) and HPV_31F2; HPV_31R2 (5’-TTGCAAACCACCTATTGGAG-3’; 5’-GATTTACCTGTATTAGGTGCACC-3’). All primers used in this study were designed based on the HPV31 prototype reference sequence (GenBank accession no J04353). To generate overlapping amplicons, Phusion® High-Fidelity DNA Polymerase enzyme mix (Finnzymes, Espoo, Finland) was utilized according to manufacturer’s instructions. Each reaction mixture contained 10 ng template DNA, 10 μl 5x HF buffer, 2.5 μl of each primer (10 μM), 1 μl 10 mM dNTPs, 0.5 μl Phusion DNA polymerase and water to a final volume of 50 μl. The amplification was carried out as follows: 98°C for 30 s, followed by 30 cycles of alternating 98°C for 5 s, 64°C or 65°C (depending on primer pair, F2_R2 or F1_R1, respectively) for 30 s and 72°C for 2 minutes 30 s. A final elongation of 5 minutes at 72°C was included. PCR products were verified by gel electrophoresis on a 1% agarose gel, purified using Promega Wizard® SV Gel PCR Clean-Up System kit (Promega, Wisconsin, United States of America) according to manufacturer’s instructions. MiSeq library preparation and sequencing was conducted at the University of the Free State Next Generation Sequencing Unit. The acquired nucleotide sequences were assembled and analysed using the HPV31 prototype(J04353) retrieved from GenBank [12].

### Phylogenetic analysis and variant lineage/sublineage identification

Comparative analysis was conducted with all 29 available complete genome sequences of HPV31 isolates from cervical carcinoma (GenBank accession numbers in S2 Table). A neighbour-joining tree was constructed with Geneious version 2019.0 (Biomatters Ltd., New Zealand), with the Tamura-Nei method. Variations within VBD13/14 were identified by alignment with the reference isolate. Transcription factor binding sites were identified within the LCR by PROMO [13, 14].

### Amplification of HPV31 LCR

Using the complete genome sequence data for VBD13/14, additional primers were designed to amplify the full-length HPV31 LCR (Table 1), to generate a fragment from nucleotide (nt) position 7069 to 180. Phusion® High-Fidelity DNA Polymerase enzyme mix (Finnzymes, Espoo, Finland) creates blunt ends, therefore, A-tailing of the PCR product was carried out by incubation of the product in 0.2 mM dATP solution (New England BioLabs, South Africa) for 30 minutes at 70°C. The PCR product was then dephosphorylated by incubation in the presence of 5 U Antarctic phosphatase (New England BioLabs, South Africa) for 30 minutes at 37°C. The reaction was stopped by incubation for 10 minutes at 70°C.

**Table 1:** Properties of primers to amplify HPV31 LCR and to conduct site-directed mutagenesis on the reporter plasmid.

Primer purpose	Primer name	Primer sequence	Annealing temperature (°C)	Position relative to HPV31 prototype (Accession # J04353.1)	Expected amplicon size
LCR fragment	HPV31_LCR F	5’-CATGTGTCTGTATGTGTATG-3’	51	7084–7103	~989 bp
	HPV31_LCR R	5’-CATCGTAGGGTATTTCCAATG-3’		180–160	
Site-directed mutagenesis	HPV31_SDM1 F	5’-TAAACTATTGTTCCTACTTGTCC-3’	53	7307–7327	~8800 bp
	HPV31_SDM1 R	5’-TAATAGTATGTTACTAATAGGGT-3’		7276–7298	
	HPV31_SDM3 F	5’-CATGCTAGTACAACTATGCTGATACAG-3’	65	7502–7528	~8800 bp
	HPV31_SDM3 R	5’-TTTAAACAATGCAACCGAAAA-3’		7501–7481	

### Construction of reporter plasmid

A β-galactosidase reporter construct was prepared using the pBlue-Topo® promoterless vector (Invitrogen, California, USA, catalogue no. K483101), with the VBD13/14 LCR amplicon containing the p97 promoter. For ligation and the following transformation into *E*. *coli* cells, the ratio of insert to vector was 1:1 and the procedure was carried out according to manufacturer’s instructions. The LCR fragment was placed upstream of the β-galactosidase reporter gene. Before conducting site-directed mutagenesis, the pBlue-Topo® LCR clones were sequenced to verify the insertion of the LCR fragment. The confirmed plasmid was designated pBlue_VBD13/14. Site-directed mutagenesis of the insert in pBlue_VBD13/14 was performed to introduce specific mutations.

### Site-directed mutagenesis

**Fig 1:**
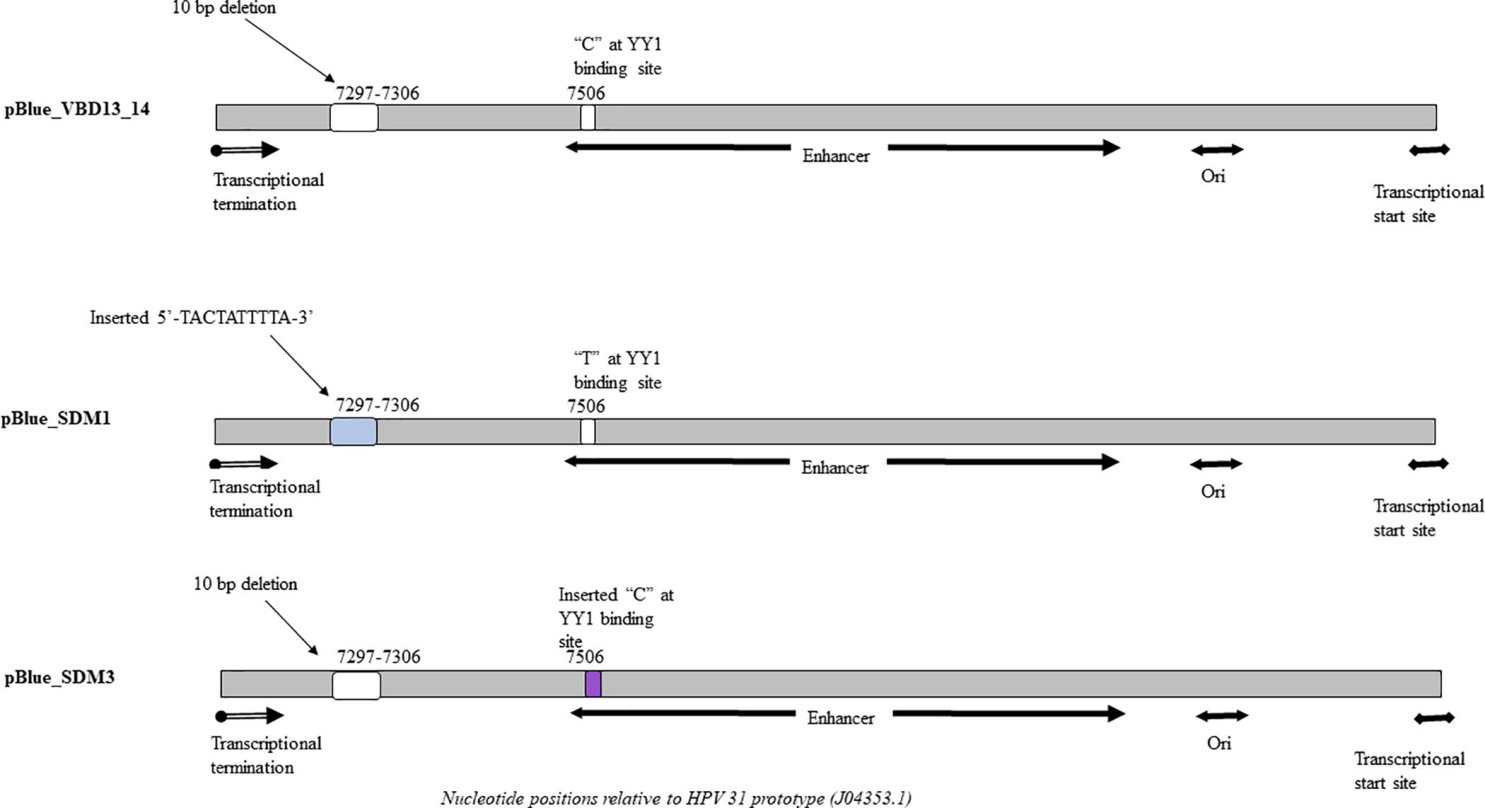
Schematic representation of the full-length LCR variants cloned upstream of the β-galactosidase gene in the reporter vector pBlue-Topo®. YY1 binding site, Yin-Yang 1 binding site; Enhancer, keratinocyte-specific enhancer domain; Ori, origin of replication of the HPV31 circular genome.

Site-directed mutagenesis by PCR was performed to introduce a specific insertion and to introduce a mutation in the LCR in pBlue_VBD13/14. A schematic representation of the full-length LCR clones is depicted in Fig 1. A 10 bp mutation absent in HPV31 lineage B was inserted (5’-TACTATTTTA-3’) using primers described in Table 1. The resultant clone was designated pBlue_SDM1. A sequence variation at a Yin-Yang 1 (YY1) transcription factor binding site, was introduced using primers containing a T-C transition. The T nucleotide is a lineage B-associated SNP, whilst lineages A and C contain a C nucleotide. This clone was designated pBlue_SDM3. No other lineage defining SNPs occurred in the transcription factor binding sites of the LCR.

Site-directed mutagenesis was performed using Phusion® High-Fidelity DNA Polymerase enzyme mix (Finnzymes, Espoo, Finland) for mutagenic primer-directed replication of both plasmid strands. The final concentration of each primer was 0.5 μM, whilst in the reaction to construct pBlue_SDM3, a final concentration of 0.13 μM was used. The basic procedure utilised the pBlue-Topo® vector with the LCR fragment insert (pBlue_VBD13/14) and two synthetic oligonucleotide primers, one of which contains the desired mutation as detailed in Table 1. The amplification profile consisted of an initial incubation at 98°C for 30 s, followed by 30 cycles of alternating 98°C for 5 s, 53°C or 65°C (depending on primers used) and elongation at 72°C for 4 minutes 30 s. A final elongation of 10 minutes at 72°C was included. Amplification was verified by separation of PCR products by electrophoresis on a 1% agarose gel.

Following thermocycling, the products were treated with *Dpn*I endonuclease, which is specific for methylated and hemi-methylated DNA to digest the parental DNA template and select for mutation-containing synthesised DNA. The reaction was set up as follows: 45 μl PCR product, 5 μl 10x *Dpn*I buffer, 1 μl *Dpn*I (New England BioLabs, South Africa) for 16 hours at 37°C. The amplicons were electrophoresed on 0.8% agarose gel and purified using Promega Wizard® SV Gel PCR Clean-Up System kit (Promega, Wisconsin, United States of America) according to manufacturer’s instructions. The purified product was eluted in 30 μl nuclease free water, dried completely at 60°C in an Eppendorf concentrator plus (Hamburg, Germany) for 15 minutes, then resuspended in 13 μl nuclease free water.

For a one-step phosphorylation and ligation, linear template (13 μl) was ligated with 1,5 μl T4 ligase, 2 μl ligase buffer and phosphorylated with 2 μl 10 mM ATP (New England BioLabs, South Africa) and 1,5 μl polynucleotide kinase (New England BioLabs, South Africa) in one reaction, for 16 hours at 4°C. Top10 *E*. *coli* cells (Invitrogen, California, USA) were transformed with the mixture, plasmid-grade DNA was extracted and a restriction digest was performed with *Bam*HI (New England BioLabs, South Africa) and *Hind*III (New England BioLabs, South Africa) to select for positive transformants. After obtaining positive clones, the constructs were validated by Sanger sequencing.

### Cell culture

Baby hamster kidney 21 (BHK) (ATCC ® CCL-10™) cells were grown in cell culture flasks in an incubator at 37°C with 95% relative air humidity and 5% CO_2_ in Dulbecco’s Modified Eagle Medium (DMEM) (Lonza, Verviers, Belgium) with 10% foetal bovine serum (FBS) (Delta products, Johannesburg, South Africa), 1% L-Glutamine (L-Glut) (Sigma Aldrich, Ayrshire, United Kingdom), 1% non-essential amino acids (NEAA) (Lonza, Verviers, Belgium), and 1% penicillin/streptomycin (Pen/Strep) antibiotics (Sigma Aldrich, Ayrshire, United Kingdom). According to their doubling time, cells were passaged every 3–5 days to keep it in the logarithmic growth phase. For passaging, the culture media was removed, and the cells were washed with 1 x phosphate buffered saline (PBS).

### Transfection of BHK cells

BHK cells were seeded at 1 x10^5^ in a 24 well plate to be 80% confluent at transfection. Untransfected BHK cells were used as negative controls. The transfection mixture contained 1 μg plasmid DNA (pBlue_VBD13/14, pBlue_SDM1 or pBlue_SDM3), 1.5 μl Lipofectamine^TM^ 3000 reagent (Invitrogen, Karlsbad, USA) and 2 μl P3000. Transfection was performed according to the recommendations of the manufacturer. Each transfection experiment was carried out independently three times.

### Transfection efficiency determined by β-galactosidase staining

Transfected cells were stained for β-galactosidase activity according to the manufacturer’s instructions (Mirus, Wisconsin, United States of America) 48 h post transfection. All reagents were provided within the kit. Staining was conducted using 5-bromo-4-chloro-3-indolyl-b-D-galactopyranoside (X-Gal) for LacZ activity. Washed cells were prefixed in 0.2% glutaraldehyde solution at room temperature for 5 min. The cells were washed three times with phosphate buffered saline (PBS) pH 7.4 and then incubated in cell staining working solution in a moist chamber and protected from light at 37°C overnight (16 h). After the incubation, the cells were washed three times with PBS and stained cells visualised using an Olympus CKX53 microscope at 10x magnification.

### Transcriptional activity measured by β-galactosidase assay

Forty-eight hours post-transfection, cells were harvested, lysed, and incubated in assay reagent according to the manufacturer’s instruction. Levels of active β-galactosidase expressed from BHK cells transfected with plasmids expressing *lacZ* gene were determined using the β-galactosidase assay kit according to the instructions of the manufacturer (Invitrogen, California, USA). The protein concentration of the cell lysate was determined using the Qubit^TM^ protein assay according to the manufacturer’s instructions (Invitrogen, California, USA). The specific activity of the cell lysate, determined in a total volume of 8 x 10^5^ nanolitres, was calculated as follows:

specific activity = nmoles of ONPG hydrolyzed/t/mg protein,

$$\mathrm{nmoles}\;\mathrm{of}\;\mathrm{ONPG}\;\mathrm{hydrolyzed}=\frac{\left({\mathrm{OD}}_{420})(8\;x\;10^{5}\;\mathrm{nanolitres}\right)}{4500\;{\mathrm{nl}/\mathrm{nmoles}}^{‐\mathrm{cm}})(1\mathrm{cm})}$$

where 4500 = the extinction coefficient, t = the time of incubation in minutes at 37°C, and mg protein = the amount of protein assayed.

For each experiment, the ratio of β-galactosidase activity of transfected BHK cells to that of the untransfected BHK cells was calculated. The ratios of relative β-galactosidase activities were compared using the independent samples t-test using SPSS version 25 (IBM Corp.).

### Ethical aspects

Written informed consent was obtained from the patient. The study was approved by the Health Sciences Research Ethics Committee of the University of the Free State (Approval no. ECUFS 137/2013D).

## Results

### Complete genome sequence and phylogenetic analysis of HPV31 VBD13/14

The first complete genome of an HPV31 isolate from detected laryngeal carcinoma in a patient in South Africa (isolate VBD13/14; Genbank Accession nr MW814876), was determined by NGS and characterised. Briefly, the complete genome of VBD13/14 was 7877 bp in length with a GC content of 37.2%. Annotation of the complete HPV31 isolate revealed eight coding regions (E6, E7, E1, E2, E4, E5, L2, L1) and two noncoding regions (intergenic E2/E5 region and LCR).

There was no sequence data available for HPV31 isolates from HNSCCs on GenBank. To determine the genetic relationship of the South African isolate and all available complete HPV31 genomes from cervical cancer from other parts of the world submitted previously to GenBank, a phylogenetic tree was constructed using Geneious version 2019.0 and classified to lineages according to Chen *et al*. [15]. (Accession numbers for 29 isolates from cervical carcinomas retrieved from GenBank are available in S2 Table).

**Fig 2:**
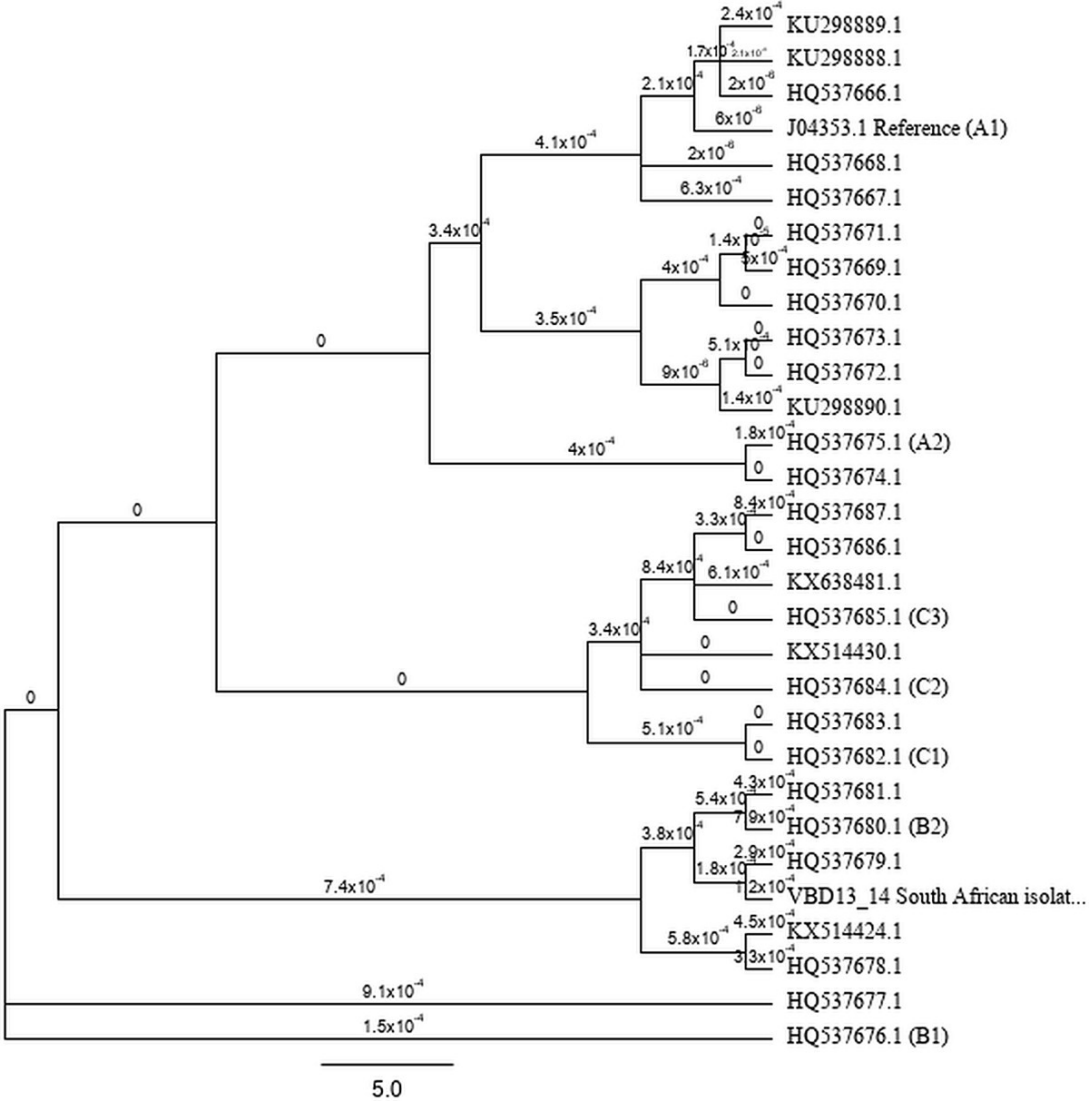
HPV31 tree topology using complete genomes. The neighbour-joining tree drawn by Tamura-Nei method was inferred from a global alignment of 29 complete sequences of HPV31 isolates from cervical carcinoma and VBD13/14 isolated from a laryngeal SCC. Labels indicate substitutions per site, there were 54 nodes and 30 tips. A bootstrap value of 100 replicates was employed. The tree was constructed with Geneious version 2019.0 (Biomatters Ltd, New Zealand). Each isolate is represented by a GenBank accession number. The representatives of variant lineages are defined by Burk et al. (2013) [8].

Isolates within lineage A formed two distinct sublineages, designated A1 and A2. Sublineage A2 included HPV31 isolates with sequences closely related to the sequence of an isolate from cervical carcinoma from Thailand (GenBank accession no. HQ537675.1). Lineage B was subdivided into two sublineages (B1 and B2) (Fig 2). Lineage C was divided into three sublineages (C1-3). The South African isolate clustered in sublineage B2 and was dissimilar to other isolates available on GenBank. However, it was most closely related to isolate HQ5376791 (Thailand) with a nucleotide sequence homology of 99.6%. As far as the intratype variability was concerned, the evolutionary divergence between variants of HPV31 lineage B (HQ537684.1 and HQ537673.1), was 1.25%.

Comparison of the 29 available complete sequences shows no distinct SNPs exclusive to VBD13/14. Sequence variations between VBD 13/14 and the reference HPV isolate were then investigated. There were a total of 59 sequence variations between VBD13/14 and the HPV31 prototype reference, comprising 40 transitions, 17 transversions and two 10 bp deletions at positions 3998 (E5 gene) and 7298 (LCR). The deletion in the E5 gene has not been described previously. Visualisation of deep sequence reads with Integrative Genomics Viewer (IGV) [16] showed that 70% of the reads contained an E5 deletion, suggesting a coinfection of HPV31 viral variants.

In order to explore natural nucleotide sequence variation in the LCR of HPV31, mutations were selected representing lineage A based on phylogenetic analysis. Altogether sixteen changes were identified in the LCR, relative to the prototype (Table 2).

**Table 2:** Sequence differences in the noncoding LCR relative to the HPV31 prototype sequence (J04353.1).

HPV 31 Prototype nucleotide position	VBD 13/14	Transcription factor binding site
7297–7306 TACTATTTTA	Deletion	-
7314–7323 TTGTTCCTAC^a^	-	-
7338 T	C	
7354 A	G	-
7372 G	C	-
7384 G	A	-
7394 C	A	-
7449–7450 GA	AC	-
7457 G	A	-
7474 C	T	-
7506 C	T	Yin-Yang 1
7525 G	A	-
7575 T	C	-
7710 C	T	-
7754 C	A	-
7865 T	G	-

^a^ 10bp deletion is a sequencing error (extra 10 bp insertion) in the prototype genome (J04353) and should not be considered a deletion [8].

### Transfection efficiency determined by β-galactosidase staining

**Fig 3:**
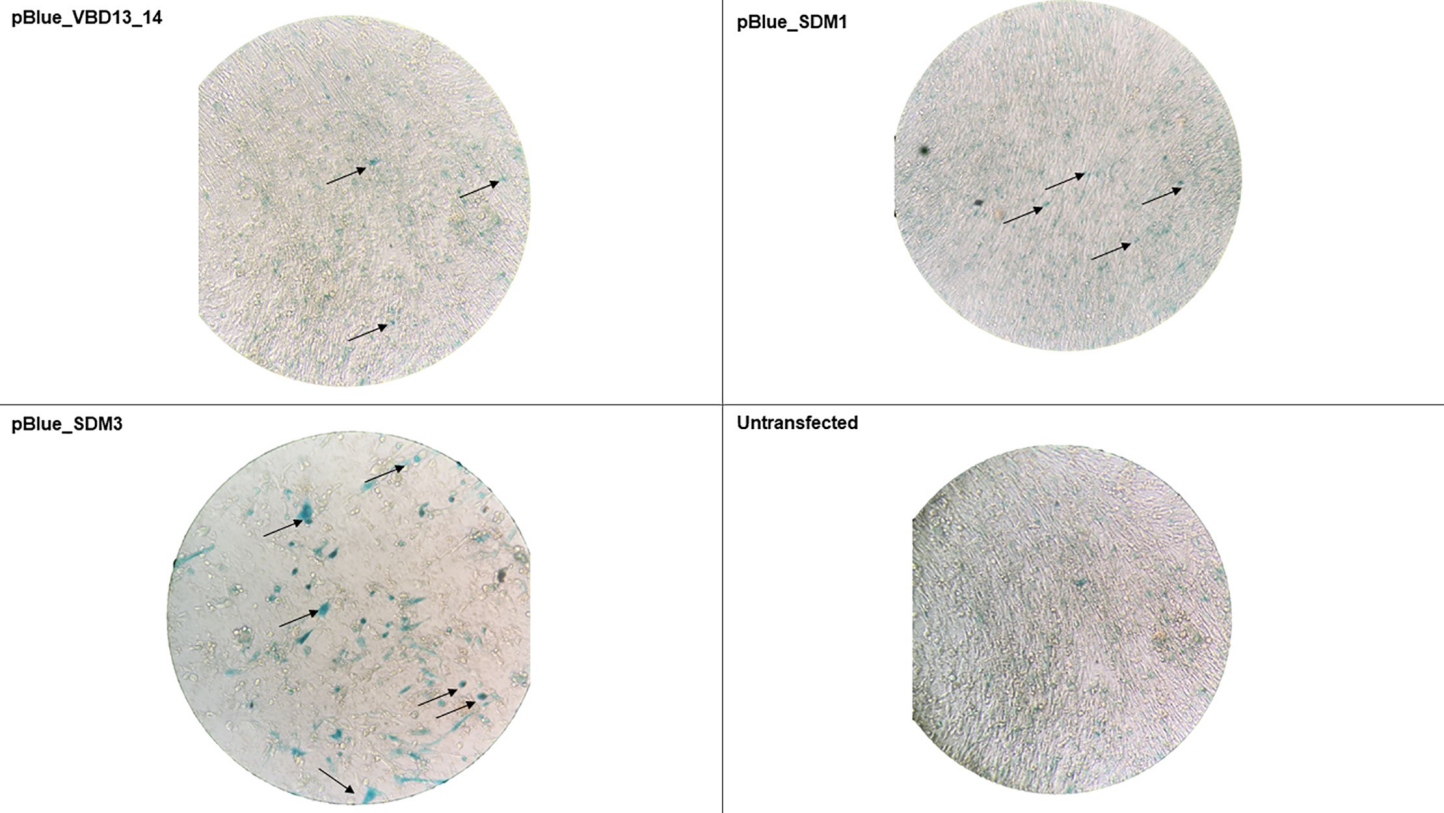
β-galactosidase staining for determination of transfection efficiency of transfected BHK cells. BHK cells transfected with pBlueVBD13_14, pBlue_SDM1 and pBlue_SDM3 and stained for β-galactosidase. Untransfected BHK cells with slight staining that shows endogenous β-galactosidase activity. Black arrows indicate some of the stained transfected BHK cells per field.

To try to localize nucleotide alterations that could potentially affect transcriptional activity, mutants were constructed to reflect changes identified relative to the prototype HPV31. These constructs were used to transfect BHK cells and the transfection efficiency of BHK cells was evaluated by X-gal staining (β-galactosidase staining kit, Mirus, Madison, WI) (Fig 3). The untransfected BHK cells showed slight staining due to endogenous β–galactosidase present in cells. This background due to endogenous staining was subsequently accounted for in the activity assays by subtracting the endogenous background.

### Transcriptional activity of HPV31 LCR mutants

**Fig 4:**
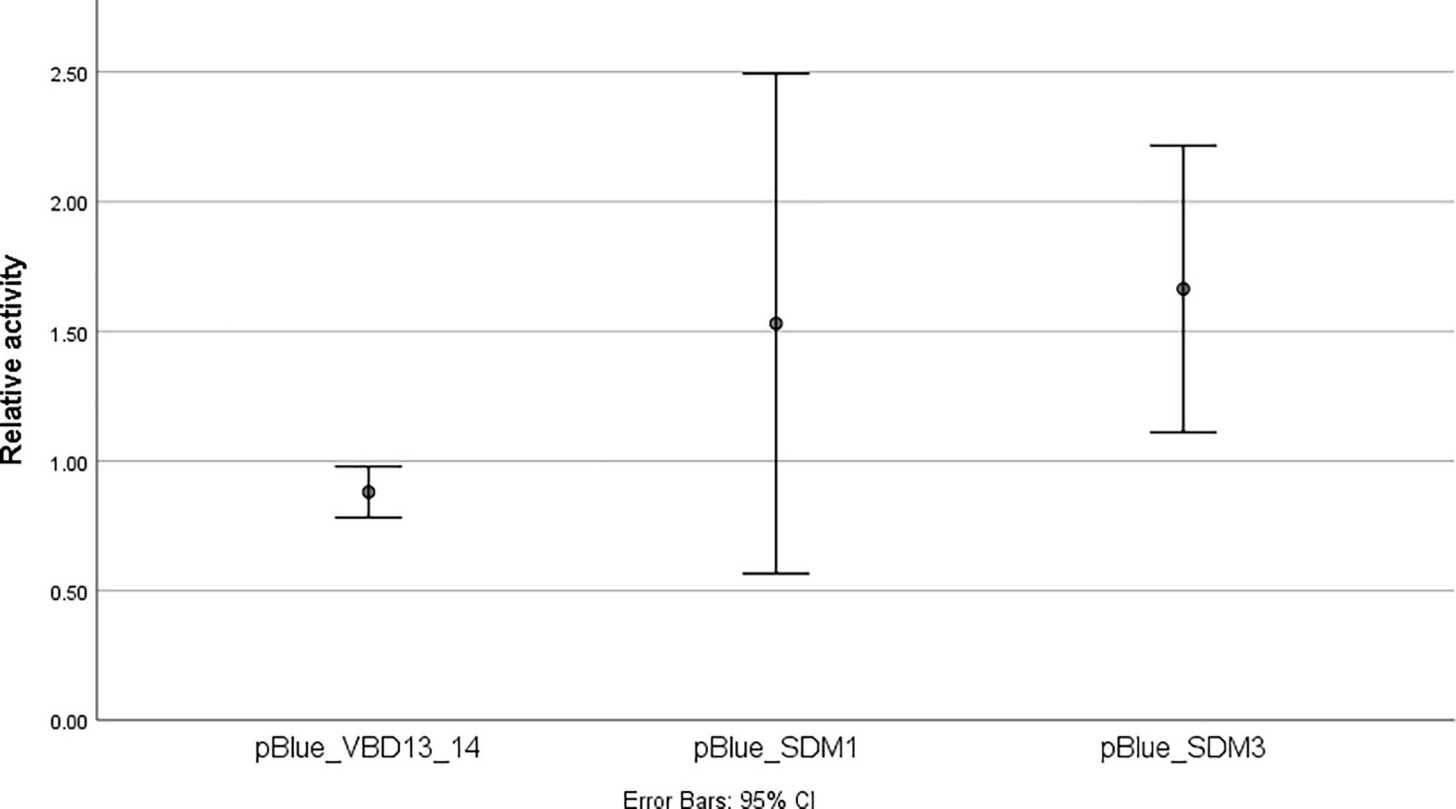
Transcriptional activity of HPV31 full-length LCR variants. Ratio of β-galactosidase activities of BHK cells transfected with reporter constructs containing different HPV31 LCR variants to that of the untransfected BHK cells.

The mean (±SD) ratio of β-galactosidase activity relative to the untransfected BHK cells was 0.88±0.09 for pBlue_VBD13/14, 1.53±0.83 for pBlue_SDM1 and 1.67±0.5 for pBlue_SDM3 (Fig 4, S1 Table). β-galactosidase activity was significantly higher for pBlue_SDM3 as compared to pBlue_VBD13/14 (p = 0.049) whereas this was not the case for pBlue_SDM1 when compared to pBlue_VBD13/14 (p = 0.251).

## Discussion

This study presents the first whole genome sequence of an HPV31 isolate from a patient with a HNSCC, adding to the 29 complete HPV31 genome sequences available on GenBank. The topology of the HPV31 phylogenetic tree confirmed the presence of three lineages. As lineage-specific SNPs were not evenly dispersed throughout the HPV31 genomes analysed, this study further supports using complete genome sequencing for HPV variant classification. Phylogenetic analysis of isolate VBD13/14 showed that the isolate clusters in lineage B. Limited nucleotide sequence divergence was observed between the three HPV31 lineages. Phylogenetic analysis shows that HPV31 acquired minor nucleotide sequence changes, which over time led to the differentiation of the lineages. Although lineage C is the most diverse, lineages A and B are more prevalent (Fig 2).

Comparison of VBD13/14 with HPV31 isolates from cervical carcinomas showed no distinct SNPs exclusive to VBD13/14. However, a novel 10 bp deletion was discovered in the E5 gene of VBD13/14. The E5 oncoprotein interacts with E6 and E7 to play an additive role in carcinogenesis [17]. It may be responsible for the stimulation of cancer cell proliferation by the formation of activating complexes with growth factor receptors such as with the Epidermal Growth Factor Receptor (EGFR), decreasing the autophagy process by downregulating the keratinocyte growth factor receptor/fibroblast growth factor receptor 2b (KGFR/FGFR2b) signalling, and promoting the accumulation of cells with abnormal DNA genetic mutations by decreasing apoptosis [17]. It also facilitates immune evasion of HPV transformed cells [16]. Future studies should extend to investigating infectivity and pathogenicity of HPV31 E5 deletion variants. The NGS data showed that 70% of the reads contained this polymorphism. These results suggest the presence of two closely related HPV31 lineage B variants in this patient. Low quality reads had been discarded before initial analysis and a high read depth was achieved, therefore this result is not a sequencing error. Co-infection with other types cannot be ruled out within this sample. Infection with another type if present would have been in very low quantities as HPV31 lineage B viral variants were the more dominant infection when genotyping this sample.

In trying to pinpoint regions of the HPV31 genome that could influence oncogenicity, this study investigated the genetically variable LCR. The LCR regulates transcriptional activity of the viral oncogenes, thus variation in this region may result in altered expression of the oncogenes. Site-directed mutagenesis used to construct variants with changes that are associated with the prototype sequence (lineage A) allowed for comparison of reporter gene activity. Increased β-galactosidase expression was observed in the mutant constructs compared to the VBD13/14 LCR, although this was not statistically significant. The highest level of β-galactosidase expression was observed with pBlue_SDM3. The C7564T change occurred at a YY1 binding site. YY1 physically interacts with several proteins regulating cell proliferation and apoptosis, including p53, Mdm2, and Rb, all of which play a role in modulating tumourigenesis [18]. In certain HPV16 isolates from cervical carcinoma [19], point mutations or deletions of YY1 binding sites in the LCR were found to have enhanced transcriptional activity [20]. The SNP and indel are representative of lineages A and C, respectively. From these results, it can be suggested that lineages A and C might have a higher expression of viral oncogenes than lineage B. In cervical cancer studies, HPV31 lineage C is more persistent than A and/or B [7, 21]. However, HPV31 lineages A/B are more commonly associated with the development of cervical intraepithelial neoplasia 3 (CIN3) [7]. Precursor lesions have not been identified in HNSCCs; therefore, it is not known which HPV31 lineages are associated with persistence or are more carcinogenic in the context of the upper aerodigestive tract.

It is important to note that variation within the LCR is not the only mechanism which leads to HPV types and variants having different oncogenic capacities. Natural sequence variation of the HPV31 E6 protein may be involved in the observed differences in the oncogenic potential between HPV31 variants [22]. In addition, combinations of amino acid changes with the oncoproteins as well as host factors may also influence oncogenic capacity.

The findings of this study are limited by the low prevalence of HPV associated with HNSCC in this population [11, 23]; comparative studies could not be completed on a large cohort of naturally occurring HPV31 variants. Further studies should create deletion mutations, to identify specific regions that are responsible for increased transcriptional activity rather than isolating single changes.

## Conclusions

This study reports the first complete genome of a South African HPV31 isolate associated with HNSCC. Results suggest that natural variation in the LCR of HPV31 isolates has a functional effect on viral p97 promoter activity. The study contributes to our understanding of the multi-factorial nature of the oncogenic potential of HPV variants and lays the groundwork for pinpointing specific genomic regions that are responsible for increased oncogenicity.

## Supporting information

S1 Table Raw data to calculate specific activity. (DOCX)

S2 Table Accession numbers of sequences used in primer design and comparative phylogenetic analysis. (DOCX)
