# Supplementary material for: Complete genome sequence of a HPV31 isolate from laryngeal squamous cell carcinoma and biological consequences for p97 promoter activity
Source: PLoS One. 2021 Aug 25;16(8):e0252524. doi: 10.1371/journal.pone.0252524 (PMC8386840; doi:10.1371/journal.pone.0252524)
Supplement: S1 Table — (DOCX) [file pone.0252524.s001.docx]

**Table S1** – Raw data to calculate specific activity.

| **Replicate** | **Plasmid construct** | **Protein concentration of cell lysate (ug/ml)** | **Protein concentration dilution factor corrected *200 (mg/ml)** | **OD420** | **nmoles ONPG** | **nmoles ONPG/min** | **Protein conc ug** | **Adjust to nmoles/min/mg** | **Specific activity** |  |
| --- | --- | --- | --- | --- | --- | --- | --- | --- | --- | --- |
|  |  |  |  | **Cell lysate volume** | **Cell lysate volume** | **Cell lysate volume** | **Cell lysate volume** | **Cell lysate volume** |  |  |
|  |  |  |  | **5** **µl** | **5 µl** | **5 µl** | **5 µl** | **5 µl** | **5 µl** |  |
| 1 | **pBlue_VBD13/14** | 14,9 | 2,98 | 0,021 | 3,73 | 0,12 | 14,9 | 67,11 | 8,35 | 0,87 |
|  | **pBlue_SDM1** | 11 | 2,2 | 0,031 | 5,51 | 0,18 | 11 | 90,91 | 16,7 | 1,74 |
|  | **pBlue_SDM3** | 11,1 | 2,22 | 0,023 | 4,09 | 0,14 | 11,1 | 90,09 | 12,28 | 1,28 |
|  | **Untransfected** | 14,8 | 2,96 | 0,024 | 4,27 | 0,14 | 14,8 | 67,57 | 9,61 | 1 |
| 2 | **pBlue_VBD13/14** | 5,28 | 1,056 | 0,041 | 7,29 | 0,24 | 5,28 | 189,39 | 46,02 | 0,97 |
|  | **pBlue_SDM1** | 2,45 | 0,49 | 0,012 | 2,13 | 0,07 | 2,45 | 408,16 | 29,02 | 0,61 |
|  | **pBlue_SDM3** | 3,15 | 0,63 | 0,038 | 6,76 | 0,23 | 3,15 | 317,46 | 71,49 | 1,51 |
|  | **Untransfected** | 1,5 | 0,3 | 0,012 | 2,13 | 0,07 | 1,5 | 666,67 | 47,41 | 1 |
| 3 | **pBlue_VBD13/14** | 13,1 | 2,62 | 0,014 | 2,49 | 0,08 | 13,1 | 76,34 | 6,33 | 0,80 |
|  | **pBlue_SDM1** | 8,04 | 1,608 | 0,024 | 4,27 | 0,14 | 8,04 | 124,38 | 17,69 | 2,24 |
|  | **pBlue_SDM3** | 10,4 | 2,08 | 0,031 | 5,51 | 0,18 | 10,4 | 96,15 | 17,67 | 2,2 |
|  | **Untransfected** | 10,5 | 2,1 | 0,014 | 2,49 | 0,08 | 10,5 | 95,24 | 7,90 | 1 |
