# Supplementary material for: Complete genome sequence of a HPV31 isolate from laryngeal squamous cell carcinoma and biological consequences for p97 promoter activity
Source: PLoS One. 2021 Aug 25;16(8):e0252524. doi: 10.1371/journal.pone.0252524 (PMC8386840; doi:10.1371/journal.pone.0252524)
Supplement: S2 Table — (DOCX) [file pone.0252524.s002.docx]

S2 Table

Table indicating accession numbers of sequences used in primer design and comparative phylogenetic analysis.

| HQ537679.1 |
| --- |
| HQ537681.1 |
| HQ537680.1 |
| KX514424.1 |
| HQ537678.1 |
| HQ537677.1 |
| HQ537676.1 |
| KU298890.1 |
| HQ537672.1 |
| HQ537669.1 |
| KU298889.1 |
| KU298888.1 |
| J04353.1 |
| HQ537666.1 |
| HQ537668.1 |
| HQ537667.1 |
| HQ537675.1 |
| HQ537674.1 |
| HQ537670.1 |
| HQ537671.1 |
| HQ537673.1 |
| KX638481.1 |
| HQ537687.1 |
| HQ537686.1 |
| HQ537685.1 |
| KX514430.1 |
| HQ537683.1 |
| HQ537684.1 |
| HQ537682.1 |
